# The Particularities of Employees’ Green Ethical Behavior in the Oil and Gas Sector

**DOI:** 10.3390/bs15010043

**Published:** 2025-01-04

**Authors:** Adriana Burlea-Schiopoiu, Camelia Olivia Timpa

**Affiliations:** 1 Faculty of Economics and Business Administration, Management, Marketing, Business Administration Department, University of Craiova, 200585 Craiova, Romania; 2 Faculty of Economics and Business Administration, Doctoral School of Economics, University of Craiova, 200585 Craiova, Romania; camelia.ilies@gmail.com

**Keywords:** code of ethics and integrity, oil and gas sector, employees’ green ethical behavior, sustainable development, ethical values

## Abstract

The article aims to evaluate the particularities of the green, ethical behavior of employees from the oil and gas sector in light of climate change and sustainable development. We employed a quantitative research approach and partial least squares structural equation modeling (PLS-SEM) using SmartPLS4 software (version 4.1.0.9) to test our conceptual model. The results demonstrate that employees’ green, ethical behavior influences how the rules of conduct contribute to an organization’s sustainable development, and employees’ green, ethical behavior is influenced by the ethical values assumed by an organization through its code of ethics and integrity. Moreover, employees’ green, ethical behavior and ethical values mediate the relationships between other factors, such as, for example, employees’ knowledge of the particularities of a company’s ethical values, their degree of compliance with the rules of conduct within the organization, and how their compliance with the rules of conduct is reflected in the organization’s sustainable development. The reflection of ethical issues in an organization’s sustainable development depends on a systemic approach to the formal and informal behavior of managers and employees. The theoretical implications of our research represent a starting point for extending our knowledge of employees’ green ethical behavior in terms of their acceptance and application of rules and ethical principles in close connection with the sustainable development of an organization. This study’s practical implications consist of awareness of the role that managers play in developing certain internal instruments (i.e., codes of ethics and integrity) to be understood and accepted by employees, with power of their personal example in motivating employees to improve their green ethical behavior. In conclusion, the manifestation of green, ethical behavior in employees does not automatically lead to sustainable development if it is not based on a set of ethical values and rules of conduct clearly understood and accepted by these employees.

## 1. Introduction

The sustainable development framework is a global objective that national and international organisms have promoted at various events. COP28 (the UN Climate Change Conference, United Arab Emirates, 30 November to 12 December 2023) concluded an agreement between more than 100 countries on transitioning from fossil fuels to renewable energy sources. The relationship between the promotion and implementation of ethical principles by organizations dealing with oil and gas production and distribution and the agreement that concluded at COP28 promotes concerted efforts to materialize sustainable development. Therefore, organizations need to increasingly focus on improving the green ethical behavior of their employees in order to reduce external pressures, which pushes them to continuously enrich their organizational culture with values that encourage employees to behave in this way (Varela-Candamio et al., 2018).

Any change generated within an organization is reflected in its reputation among external stakeholders (Balan & Burlea-Schiopoiu, 2017). Therefore, the importance of implementing ethical principles in large companies has been studied by researchers and practitioners, especially in the context of ethical scandals that have targeted companies from various fields of activity, such as Silicon Valley Bank (Santa Clara County, CA, USA) (Shane & Mahi, 2023) and Boeing Co. (Arlington County, VA, USA) (Schaper, 2022).

Starting from the premise that oil and gas sector organizations cause negative effects on the environment through their activity, effects that leave their mark in terms of climate change, we believe that a responsible internal approach to the principles of sustainable development must be based on clear rules that are known and understood by all employees. Therefore, an ethical internal climate will lead to real awareness in employees of the risks to which both the organization and the community are exposed through releasing toxic products into the atmosphere (for example, by flaring gases). Social responsibility and business responsibility are determining factors of an organization’s survival, and effective merging of these responsibilities can be achieved through the active involvement of employees. The practical mechanisms of sustainable development must be based on ethical norms and principles that act effectively to avoid the polluter pays principle, and they must be contained in a code of ethics and integrity that promotes the equal rights, obligations, and responsibilities of employees.

Hendy and Tucker (2021) believe that failure, especially the ethical failure, of organizations is the result of the tacit collective acceptance of the violation of ethical principles by most employees. Paradoxically, in some organizations, the manifestation of unethical behavior is either ignored or is justified with various arguments, such as invoking the limited knowledge that employees have about the particularities of the ethical values within the organization or giving little importance to the ethical values assumed by the organization through the code of ethics and integrity for employees.

Employees justify their unethical behavior with their preferred and dominant personal values, which converges with a lack of ethics (Pang et al., 2022; Ripoll & Schott, 2020). To prevent unethical behavior from manifesting in organizations, a series of legislative acts regulate the codex of conduct for employees. Therefore, the objective of our study is to identify the factors that influence the ethical behavior of employees in organizations in a field that has a direct impact on sustainable development (oil and gas sector organizations) and to emphasize the importance of awareness of the ethical dimension of sustainable development for organizations, regardless of whether they are public or private.

In this context, to draw sustainable ethical coordinates in oil and gas sector organizations, it is necessary to investigate how employees perceive the impact of moral values and norms on their commitment to their organization and the community.

A green psychological climate can be fostered by effectively implementing the ethical values outlined in a code of ethics (Dumont et al., 2017; Norton et al., 2017; Zatonatska et al., 2024). Green psychology is not just a theoretical concept but a complex and practical one that involves aligning organizational policies with employees’ work procedures (Ramus & Steger, 2000; Zhou et al., 2018). This is the foundation of our following research questions:

- Does the organization’s code of ethics serve as a practical tool for enhancing the interaction of employees with their work environment by shaping specific patterns of organizational practices and policies?
- How can oil and gas sector organizations promote employees’ ethical responsibilities in agreement with sustainable development responsibilities?

In oil and gas sector organizations, employees’ ethical and environmental values should be included in the evaluation process, which will increase their awareness and knowledge of these values. That is why employees must be trained and then involved in putting into practice the rules of the code of ethics that lead to the promotion of green ethical behavior.

To the best of our knowledge, no research has been conducted to analyze how knowledge of and adherence to oil and gas sector organizations’ ethical values are reflected and perceived by employees as promoting sustainable development. Therefore, we believe that our study will contribute to the conceptualization of the ethical values of organizations involved in the production and distribution of oil and gas from the perspective of the moral status of all employees, both those with management functions and those with execution functions.

## 2. Literature Review and the Development of the Theoretical Model

Employees’ green ethical behavior is complex behavior implemented in the DNA of human beings and manifests itself through permanent concern on the part of individuals for caring about sustainable development and respecting ethical principles. The working environment offers employees the opportunity to manifest their green ethical behavior in an organized environment while providing them with motivational elements that lead to the development of this type of behavior.

The manifestation of green ethical behavior is coordinated through the power of personal example, which eliminates the line of demarcation between management functions and execution functions. Thus, green ethical behavior is guided by ethical and environmental values but is not limited to a certain territory.

Organizations that create the right environment for their employees to manifest their green ethical behavior will have a competitive advantage because this type of behavior leads to the development of affective commitment in employees. In one way or another, by encouraging employees’ green ethical behavior, organizations help employees to make rational decisions (which is reflected in Ajzen and Fishbein (1980)’s theory of reasoned action—TRA), plan their behavior according to the pressing challenges of the moment (which is reflected in Ajzen (1991)’s theory of planned behavior—TPB), be guided by a personal and organizational set of values, beliefs, and norms (according to Stern (1999)’s value-belief-norm theory—VBN), and self-coordinate through the interrelationship between organizational, personal, and environmental factors (which is in agreement with Bandura’s (1986, 1989) social cognitive theory—SCT).

### 2.1. The Manifestation of Employees’ Green Ethical Behavior in the Context of Compliance with the Rules of Conduct That Are Reflected in the Sustainable Development of the Organization

The ethical challenges for oil and gas organizations in fields that have a direct impact on sustainable development lead to other challenges based on the relationship between the ethical values of an organization and sustainable development, such as the relationship between the ethical values assumed through the code of ethics and integrity and the commitment of employees to an organization and the relationship between an organization’s ethical values and how compliance with the rules of conduct is reflected in the process of sustainable development, not only of the oil and gas sector organization but also of the community in which it operates (Burlea-Schiopoiu & Mihai, 2019).

Therefore, we can start from the premise that an organization’s ethical values can leave their mark on employees’ decisions regarding the ecological impact that their activities may have on the environment. In demonstrating green ethical behavior, employees will pay more attention to ethical principles if their activity is considered to have a negative ecological impact on the environment. Thus, green ethics practices in oil and gas sector organizations must be transparent and become a priority with visible activities both in the organization’ internal environment (i.e., ensuring safe working conditions; paying more attention to reducing energy consumption and reducing waste; programs of training oriented towards sustainable development practices) and in its external environment (i.e., the use of efficient and non-polluting technologies; the implementation of programs to reduce carbon emissions; the protection and conservation of habitats).

Numerous studies have been dedicated to employee green behavior (Dumont et al., 2017; Guerci et al., 2016), but the specialized literature faces a gap in terms of researching the complex relationships between green and ethical behavior in the context of sustainable development by means of internal organizational tools (i.e., ethical codes). Moreover, Raineri and Paill (2016) emphasize the need to carry out empirical studies that analyze the impact that compliance with the rules of conduct within an organization has on its employees’ green, ethical behavior and the organization’s sustainable development. Therefore, sustainable development objectives must be correlated with the principles of the code ethics and found in the organization’s ecological practices.

In this regard, we define employees’ green ethical behavior (EEGB) as behavior that uses an organization’s ethical principles and values as a tool to strengthen itself. Moreover, EEGB can have positive effects on the organization and contribute to its sustainable development (RCSD), especially if it is also used as an instrument to increase the organization’s reputation.

Rehman et al. (2023) concluded that organizations must develop and implement a proactive environmental strategy to increase their ecological performance. This strategy also contributes to developing green, ethical behavior among employees. As a result, the code of ethics is a relevant tool that guides the green, ethical behavior of employees because among the values indicated in the code of ethics are honor, transparency, and responsibility. Therefore, these values may lead employees to feel responsible for preserving the environment for the next generation and to develop their sense of responsibility for the environment. That is why in the context of sustainable development, the code of ethics must continuously evolve and explicitly include provisions that guide employees’ green, ethical behavior by promoting ecological, ethical actions that underline the importance of the ethical values assumed by the organization through the code of ethics and integrity of employees (IEV).

Ethics advisors have the role of championing ethical values among employees by informing them about the existence and application of rules of conduct within the organization. Importantly, the internal code of ethics should explicitly state that employees are protected from any form of sanction or prejudice if they seek advice from the ethics advisor on compliance with the principles and rules of conduct or if they report violations of the ethical principles by other employees. This provision ensures a safe and secure environment for employees to uphold ethical standards and aims not only to improve the organizational reputation but also to actively engage in sustainable development. By promoting ethical behavior, organizations demonstrate their commitment to societal values and their role in fostering sustainable business practices.

Implementing ethical principles in organizations is essential to creating a favorable work climate and reducing the possibility of ethical scandals. Therefore, employees must have a solid knowledge of an organization’s ethical values (EKE) and be guided in their activities by the norms of the organization’s code of ethics and integrity (IEV).

The governance structure, built on ethical principles, is not just a set of rules but a guide to responsible behavior. By making employees aware of the legal measures that can be taken if they violate the rules of conduct, an organization provides a sense of security and guidance, enhancing confidence in the ethics advisor and reassuring employees of the organization’s commitment to ethical practices.

Moreover, managers must be an example for their employees in promoting sustainable development actions internally and in relations with stakeholders, thus creating an organizational culture that includes sustainable development practices. As a critical analysis of the literature, we formulated the following hypotheses.

**H1:**  *Employees’ green ethical behavior (EEGB) directly influences how compliance with the rules of conduct is reflected in an organization’s sustainable development (RCSD).*

**H2:**  *Evaluation of employees’ degree of knowledge of the particularities of the ethical values manifested in an organization (EKE) directly influences employees’ green ethical behavior (EEGB).*

**H3:**  *Evaluation of employees’ degree of knowledge of the particularities of the ethical values manifested in the organization (EKE) directly influences the importance of the ethical values assumed by the organization through the code of ethics and integrity of employees (IEV).*

### 2.2. Tools That Influence the Ethical Values of Organizations and Contribute to Sustainable Development

The code of ethics is a powerful tool that can shape employees’ green, ethical behavior due to its potential, which can only be fully realized when a strong bond is formed between management, employees, and the ethics committee or integrity advisor. Therefore, each of these actors plays a specific role in upholding ethical values, making them feel the importance of their roles in an organization’s sustainable development.

Schwartz (2002) emphasized the importance of implementing the code of ethics by enhancing moral guidelines, which lessens the danger of a code of ethics being downplayed and losing the message it wants to convey to employees. Each organization has several branches, but the employees in these branches perceive the content of the code of ethics differently. Therefore, even if the code of ethics is developed at the top level of the organization, it must be distributed to each branch, subject to the addition or removal of elements that are not accepted by the employees in one branch or another.

Trevinyo-Rodriguez (2007) focused his attention on the trait of integrity and observed that this characteristic has different connotations depending on the particularities of the domain to which it refers. That is why, for employees to manifest ethical behavior, it must be based on moral decisions, especially in oil and gas sector organizations, and it is necessary to continuously evaluate the degree of compliance with the rules of conduct within an organization (RCC).

Helin and Sandström (2010), as a result of an analysis of how subsidiary employees understand and implement the code of ethics developed at the organizational level, concluded that even if the members of the subsidiary do not agree with or do not understand some of the rules set out in the code of ethics, they state that they have understood the code of ethics and fully agree with it. That is why, through our study, we also evaluate whether in the case of Romanian oil and gas sector organizations, this behavior of positioning outside of power is confirmed and the ethical norms and values included in an organization’s code of ethics and integrity are unconditionally accepted.

According to Benson (1989), companies’ codes of ethics are subject to a cyclical process of updating depending on the challenges that appear in the market and the scandals that manifest themselves at the national or international level. Therefore, employees must be informed about a company’s ethical values and about any changes in its code of ethics so that the norms of conduct are respected and they are concerned about the application of these values and ethical norms in the context of sustainable development. The ethical behavior of managers is a benchmark for employees because if managers show contempt for the company’s ethical values, employees will also show the same behavior. For example, suppose that managers arrogate to themselves salary rights or other advantages that other employees in the company do not benefit from. In this case, employees will seek to obtain similar advantages by violating moral norms and ethical principles, motivated by the managers’ behavior. That is why evaluation of employees’ performance must include a section on how these employees apply ethical norms.

**Figure 1:**
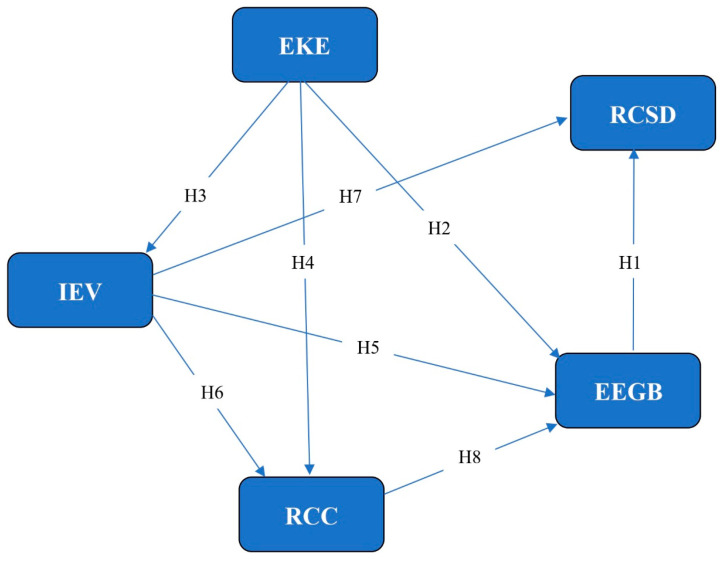
Theoretical model of the research.

Synthesizing the specialized literature, we formulated the following hypotheses and developed the research model (Figure 1):

**H4:**  *Evaluation of employees’ knowledge of the particularities of the ethical values manifested in an organization (EKE) directly influences the evaluation of the degree of compliance with the rules of conduct within the organization (RCC).*

**H5:**  *The importance of the ethical values an organization assumes through the code of ethics and integrity of employees (IEV) directly influences employees’ green ethical behavior (EEGB).*

**H6:**  *The importance of the ethical values assumed by an organization through the code of ethics and integrity of employees (IEV) directly influences the evaluation of the degree of compliance with the rules of conduct within the organization (RCC).*

**H7:**  *The importance of the ethical values assumed by the organization through the code of ethics and integrity of employees (IEV) directly influences how compliance with the rules of conduct is reflected in an organization’s sustainable development (RCSD).*

**H8:**  *Evaluation of the degree of compliance with the rules of conduct within an organization (RCC) directly influences employees’ green ethical behavior (EEGB).*

The proposed research model (Figure 1) evaluates the direct effects on the green ethical behavior of the employees and the sustainable development of oil and gas sector organizations, as well as mediations between the variables.

## 3. Methodology

### 3.1. Research Design and Measures

In our research, we carefully chose a quantitative approach. Our theoretical model’s complexity and robustness required a higher-order construct, which led us to use partial least squares structural equation modeling (PLS-SEM). Compared to other statistical methods (e.g., CB-SEM or multiple regression), PLS-SEM incorporates more advanced methods, including a bootstrapping technique or weighted composites of indicator variables, providing greater statistical power (Hair et al., 2022).

We developed our conceptual framework based on a structured questionnaire, which was divided into six comprehensive sections.

The first section presents details about the respondents, such as their gender, age, category (i.e., execution function or management position), total seniority in the work, and seniority in the organization. The second section is dedicated to employees’ green ethical behavior (EEGB) and contains four items adapted from Bissing-Olson et al. (2013), Iqbal et al. (2018), and Raineri and Paill (2016). The third section deals with the evaluation of the employees’ degree of knowledge of the particularities of the ethical values manifested in the organization (EKE) and includes three items adapted from Jensen et al. (2015). The fourth section has six items and refers to the importance of the ethical values assumed by the organization through the code of ethics and integrity for employees (IEV), which is adapted according to Trevinyo-Rodriguez (2007). The fifth section evaluates the degree of compliance with the rules of conduct within an organization (ECC) and contains eight items adapted from Adams et al. (2001). The last section aims to assess how compliance with the rules of conduct is reflected in sustainable development (RCSD) and contains three items adapted from Chou (2014).

The questionnaire items distributed online to employees provided evaluations using a five-point Likert scale from 1 = strongly disagree to 5 = strongly agree. To ensure that all of the variables were familiar and easily rated by the respondents, a pretest of our measures was conducted on 12 respondents with managerial positions and 40 respondents with executive positions. Based on these respondents’ feedback, we proceeded to refine the content of the items on the variables related to the importance of the ethical values assumed by an organization through the code of ethics and integrity for employees and the degree of knowledge of the employees of the particularities of the ethical values manifested in the organization.

### 3.2. Sample Selection

This study was conducted in Romanian oil and gas sector organizations. In sustainable development, the production and distribution of natural gas are given increased attention, especially since, according to COP28, a global transition from fossil fuels to renewable energy sources is being pursued.

The sample size of 319 employees was thoughtfully selected from oil and gas sector organizations that, through their field of activity, are committed to using environmental sustainability practices in line with the agreement that concluded at COP28.

The questionnaire was pretested on a sample of 10 managers and 50 employees to assess how the respondents assessed the reliability of the variables and items. The questionnaire was distributed online, and the Ethics Officer provided the information between January and March 2022. The results helped us to adjust the wording of some items so that they were more appropriate for our research.

The second stage of the research was carried out between September and December 2022, with 403 questionnaires being completed, of which 319 were valid, representing an effective response rate of 79.16%.

Since the questionnaire was distributed online, the respondents could opt out of completing the questionnaire at any stage. Thus, according to the preliminary information provided before the questionnaire began, the respondents tacitly gave their consent when they started the questionnaire but especially when they completed the questionnaire, which took between 15 and 20 min to complete.

We note that the “Respondent Data” section included general questions (gender, age, seniority in the organization) that did not allow for the precise identification of the respondents, precisely in order to protect their confidentiality and respect their rights. Finally, our study constructs were tested for common method bias right from the design phase and then in the pretesting phase of the questionnaire to avoid ambiguities and to formulate the items so that they were as simple as possible for the respondents to understand, according to the recommendations of Podsakoff et al. (2012) to use a procedural technique and to control method biases through complex statistical techniques.

Table 1 not only provides a detailed breakdown of the sample but also incorporates descriptive statistics, thereby further fortifying the robustness of our methodology.

Analyzing the above table, we observe that the preponderance is men (76.5%). This is a consequence of the field of activity being less accessible to women than men due to difficult working conditions. Another worrying observation refers to the low proportion of young people (16.3%), which represents both a topic for reflection for managers and a concern for the human resources management of these organizations.

## 4. Results

Table 2 presents certain elements (i.e., the outer loading, Cronbach’s alpha, composite reliability values, and average variance extracted (AVE) values for all of the variables and their items) necessary for evaluating the model’s reliability.

The data prove that the item loadings of the indicators in this study range from 0.723 to 0.951, and all of them are above the threshold; the values of the Cronbach’s alpha ranges from 0.881 to 0.944, and the values on the composite reliability (CR) range from 0.884 to 0.955, with all exceeding the threshold value of 0.700 (Hair et al., 2022). In terms of the average variance extracted (AVE), the values range from 0.663 to 0.894 and are above the threshold value of 0.500 (Fornell & Larcker, 1981). Therefore, the values prove that convergent validity is ensured.

The discriminant validity was evaluated according to the heterotrait–monotrait ratio of the correlations (HTMT), and we observed that problems related to discriminant validity did not exist because the highest value of the HTMT was 0.692, below the threshold (0.85) recommended by Henseler et al. (2015).

According to Henseler et al. (2015), the value of the standardized root mean residual (SRMR) was 0.079, lower than the threshold value of 0.08, and the normed fit index (NFI) was 0.916, above the value recommended, at 0.9. Therefore, the values of these two indicators prove a good model fit. The model does not show multicollinearity issues because the variance inflation factor (VIF) values were less than 5.0 (Chatterjee & Simonoff, 2013).

The hypotheses were tested using a bootstrapping process (10,000 bootstrap samples), using a 95% bias-corrected confidence interval and a two-tailed test (Table 3).

**Figure 2:**
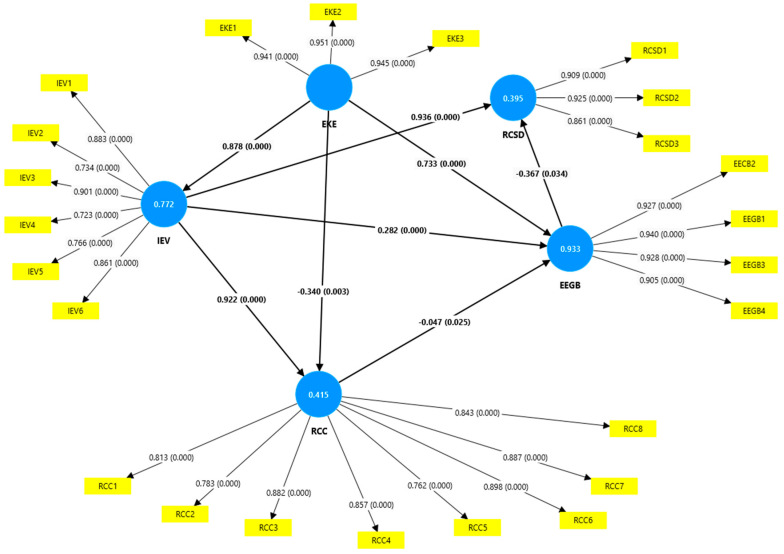
The hypothesis testing. Source: Smart PLS software: SmartPLS.

Figure 2 depicts the validation of the relationships between the variables in the research model.

From the analysis of Table 3 and Figure 2, we notice that all of the hypotheses were validated, which entitles us to affirm that an organization’s ethical values and norms must be presented in the organization’s code of ethics in a way that allows all employees to understand the mechanisms through which they can be respected and implemented. Moreover, it confirms the need for employees to know the ethical values manifested in an organization to establish them and prioritize them according to the specifics of their activity. Therefore, Table 4 depicts the mediating relationships between variables.

Delving into the intricate mediation relationships between variables, it becomes evident that employees’ green, ethical behavior is a multifaceted factor that not only influences but is also influenced by other organizational factors.

The green, ethical behavior of employees mediates the relationship between their degree of knowledge of the particularities of the ethical values manifested in a company and their observance of the rules of conduct that are reflected in sustainable development (EKE -> EEGB -> RCSD; *p* = 0.032) because EEGB contributes to their awareness regarding the need to know the ethical principles and values in the code of ethics in order to create and respect the health and safety regime at work, thus creating the premise for them to perform professional tasks in a way that positively affects the environment.

Green ethical behavior does not mediate the relationship between the importance of the ethical values assumed by a company through the code of ethics and integrity for employees and how compliance with these rules of conduct is reflected in sustainable development (IEV -> EEGB -> RCSD; *p* = 0.060) because employees believe that in fulfilling their professional tasks and by respecting the values promoted by the company’s code of ethics, they must carry themselves in a way that does not affect the environment and is in line with their ethical values.

Furthermore, employees believe that freedom of expression, equal opportunities and treatment, transparency, objectivity, and impartiality can lead to responsible use of resources and awareness of the importance of developing green ethical behavior that directly impacts the work environment and their affective commitment.

It is not surprising that EEGB does not mediate the relationship between how the rules of conduct are respected within a company and how the respect for the rules of conduct is reflected in sustainable development (RCC -> EEGB -> RCSD: *p* = 0.211). Employees perform their professional tasks and respect the company’s ethical code in a way that positively affects the environment because they care about the environment both now and for future generations. However, this behavior agrees with their professional responsibilities, the responsible use of resources, and the manifestation of absolute objectivity and responsibility in making ecological decisions. Therefore, green ethical behavior is in agreement with a company’s ethical rules and values and not a mediating factor because employees believe that by implementing these ethical principles and values, they make an important contribution to raising the awareness of colleagues and other members of the community of the importance of green ethical behavior both at work and in all of their daily personal activities.

The importance of EEGB to companies and to the communities in which they operate also results from the fact that good knowledge in the employees of the particularities of the ethical values manifested in a company strengthens EEGB but is not mediated by compliance with the rules of conduct within the company (EKE -> RCC -> EEGB; *p* = 0.105). Therefore, we can state that compliance with the rules of conduct within a company is considered a sine qua non principle because the green ethical behavior of employees encompasses extensive responsibilities that manifest themselves both within the company and in their families and the community. Moreover, compliance with the rules of conduct within a company (IEV -> RCC -> EEGB; *p* = 0.039) mediates the relationship between the importance of the ethical values assumed by the company through the code of ethics and integrity for employees and EEGB in the sense that these ethical values must be defined and adjusted according to the result, namely according to the awareness by employees of the need for EECG both for professional development and especially for personal development.

Therefore, the importance of the ethical values assumed by a company through its code of ethics and integrity for employees also results from the mediating relationships between the degree of knowledge of the employees of the particularities of the ethical values manifested in a company and three other variables, such as EKE -> IEV -> RCC, *p* = 0.000; EKE -> IEV -> RCSD, *p* = 0.000; and EKE -> IEV -> EEGB, *p* = 0.000.

Analyzing these three mediating relationships, we observe that the ethical values that companies assume leave their mark on all of the actions and behaviors of employees because any behavior is guided and manifests itself according to what the company promotes as ethical values and principles. Beyond these values and principles, the behavior of the employees is guided by their personal values, which, in some cases, may conflict with the company’s values, which can cause the whistleblowing phenomenon.

## 5. Discussion

Our findings state the interdependence between employees’ degree of knowledge of the particularities of the ethical values and compliance with an organization’s rules of conduct, which agrees with the findings of Harrison et al. (2018), who considered that psychological factors strongly influence employees’ unethical behavior, especially in the absence of a code of ethics unanimously accepted and known by these employees.

Jensen et al.’s (2015) research underlines that successfully implementing the values and norms of an organization’s code of ethics depends on responsible actions being taken by employees and managers, which implies a lower level of control. Therefore, increased responsibility and reduced control are two attributes that contribute to compliance with the rules of conduct and the development of employees’ green ethical behavior. Moreover, Haunstrup and Jensen (2024) and Burlea-Schiopoiu (2007) found that managers, by using effective communication oriented towards objectives, especially sustainable development objectives, actively contribute to increasing the efficiency of training programs, which can lead to the manifestation of green ethical behavior among their employees.

The validation of the hypotheses confirms the importance of the relationship between the ethical values assumed by an organization and employees’ compliance with its rules of conduct, which impacts sustainable development. In this sense, sustainable development responsibilities must be well defined for personnel in management and execution functions. Through decision-making power, management staff can be more involved in the process of implementing ethical values and norms that have long-term effects on the sustainable ethical behavior of their employees by creating the right framework for reducing sanctions as much as possible but also through recognition of employees’ rights. This is why we recommend that codes of ethics for oil and gas sector organizations contain detailed explanations of certain rules but especially details regarding sanctions, so that all employees, regardless of whether they are involved in execution or production, have very clear principles based on which the organization’s norms and values are consolidated.

The role of ethics whistleblowers should not be neglected. The whistleblower must be objectively involved in resolving complaints from employees, especially those from employees in executive positions. Whistleblowers, if they fail to communicate effectively with employees, can turn the positive effects of a code of ethics into negative effects, with direct repercussions on how employees perceive their individual ethical responsibility (Clegg et al., 2007).

Therefore, the development and implementation of ethical principles must be carried out through the participatory collaboration of all stakeholders because in this way, the quasi-existent resistance from employees who either do not understand the content of the code of ethics or do not agree with specific provisions of the code can be avoided (Norton et al., 2017). Magni et al. (2024), based on a systematic literature review, concluded that organizations can achieve a competitive advantage by promoting and developing ethical employee behavior. Moreover, the interaction between various theories (e.g., stakeholder theory, SCT, TPB) is positively reflected in a business strategy that allocates a generous space to ethical employee behavior. Moreover, according to Hauser (2020), the validation of the relationships between EKE and IEV, on the one hand, and between EKE and RCC, on the other hand, demonstrates the importance of correlating the ethical values and principles from the code of ethics and integrity with the activities of the employees in order to induce them to implement practical green behavior oriented towards the organization’s sustainable development.

Codes of ethics are tools that can contribute either to an improvement in managers’ control systems or to the creation of a work climate based on stimulating communication between employees at all levels in which, in real time, violations of ethical values can be reported without them being afraid of sanctions following the resolution of the referral or of being accused of whistleblowing.

Adelstein and Clegg (2016) believe that the code of ethics somewhat forces employees to accept a company’s rules as a sine qua non because the aim is to establish a connection between the company’s interests and the employees’ ethics through the code of ethics, which is the legal instrument of control of the ethical behaviors of employees. Furthermore, Husted’s (2005) codes of ethics manage strategic risks by clearly demarcating ethics from employees’ unethical behavior and sanctioning any violation of their principles.

Change comes from within an organization. Therefore, the green, ethical behavior of employees who, through their activity, have a direct impact on environmental protection is a mediating factor between the need for employees to quickly find answers to their ethical dilemmas and how an organization, through its rules of conduct, promotes sustainable development both among its employees and in the community (Abbas & Dogan, 2022; Shahzad et al., 2023). Tzanidis et al. (2024) established positive correlations between organizations’ green competitive advantage and the integration of environmental objectives into employee behavior strategies. Green marketing is a concept that encompasses elements other than advertising that lead to employees’ green ethical behavior.

In agreement with Lalot et al. (2019) and Kim et al. (2017), our findings prove that EEGB does not mediate the relationship between IEV and RSCD (*p* = 0.060) nor the relationship between RCC and RCSD (*p* = 0.211) because EEGB is very much dependent on personal ethical beliefs and values and IEV, RCC, and RCSD are variables that have elements in their structure that mainly depend on how ethical dilemmas are managed (i.e., the code of ethics incorporates an organization’s values as desired by the management and not as a result of consulting all employees, which is why organizations are faced with dilemmas regarding the degree of acceptance or ignorance of the rules of their codes of conduct and the problematic manifestation of irresponsible ethical behavior by their employees) and those related to sustainable development.

Qalati et al. (2024) highlighted the role of green human resource management practices in senior management’s commitment to sustainable development, which increases an organization’s environmental performance. After analyzing senior management’s green ethical behavior, we focused on how employees perceive the need to hone their roles according to their personal, organizational, and community values.

How Romanian society perceives the ethical behavior of employees depends a lot on the ethical principles promoted inside the organization and on the communication of these principles in the organization’s external environment. Therefore, our findings prove that it is essential for employees to be aware of the need to create a green ethical climate by putting into practice the values and principles of the code of ethics with the support of an ethical advisor. This will create and develop a green ethical spirit inside the organization.

## 6. Conclusions, Theoretical and Practical Implications, Limitations, and Future Research

COP28, by promoting a gradual transition to renewable energy sources, launched an important ethical challenge for organizations involved in the extraction, processing, and distribution of fossil fuels. Therefore, the organizations involved in this process must approach the promotion and implementation of ethical values among its employees from the perspective of integrating the individual well-being of the employees with the need to ensure a healthy environment for future generations through active involvement in protecting the environment.

Understanding an organization’s ethical values and, more importantly, recognizing their significance to employees are relevant. This understanding can drive the ethical motivations behind the economic decisions made at the organizational level. When taken in the interest of both employees and the community, such decisions can lead to more sustainable and ethical business operations.

Research on the role of ethics committees in promoting ethical values has been oriented primarily towards the fields of education (Brown et al., 2020) and medicine (Noble, 2017) and less towards public organizations, which, through the objects of their activities, are directly involved in reducing pollution and protecting the environment. This is why through our research, we have filled a gap both in the literature, by critically analyzing the aspects related to green and ethical behavior, and in practice, by evaluating the impact of ethics not only on an organization’s climate but also on the community and the environment in which organizations operate (Hong et al., 2023).

### 6.1. Theoretical Implications

Little research has focused on how employees become involved directly and with tangible results in developing their organization’s code of ethics (Collinson, 1994; Helin & Sandström, 2010). In fact, until now, no studies have been carried out regarding the factors that lead to the production of visible effects with an impact on green, ethical behavior in employees, such as the manifestation of an ethical climate in organizations, how the implementation of a new code of ethics leads to positive changes in the behavior of employees, and whether the code of ethics is considered a decorative document or a tool that actually supports employees to solve their ethical dilemmas.

Most of these studies intended to analyze the factors that determine unethical behavior in employees (Sheedy et al., 2021; El-Haber et al., 2024), neglecting precisely the factors that favor the manifestation of ethical behavior. This is why the incorporation of control into organizations’ codes of ethics is considered a strategy that aims to cause employees to orient themselves towards ethical behavior and to eliminate or reduce their temptation to behave unethically.

Our research places significant emphasis on the role of employees in shaping the ethical behavior of oil and gas sector organizations. Our findings underscore the importance of ethics tools and programs in oil and gas sector organizations meeting several conditions to stimulate ethical behavior in their employees. These conditions include them being developed with the input of employees, being clear enough to avoid any possibility of a tendentious or personal interpretation, and being known and unanimously accepted by the employees, regardless of their hierarchical level. This approach not only values the input of employees but also recognizes their integral role in the ethical development of organizations.

Finally, we tested and validated a new model that can serve as a catalyst for researchers to develop innovative methods and models for strengthening the relationship between the ethical values of oil and gas sector organizations and sustainable development. This model, based on our findings, has the potential to revolutionize the way oil and gas sector organizations approach ethics and sustainable development. Our study not only contributes to the existing literature but also paves the way for a more integrated understanding of the ethical values of oil and gas sector organizations and how employees perceive the usefulness of these ethical values in relation to sustainable development.

### 6.2. Practical Implications

Our study represents an opportunity for decision-makers, both for those in oil and gas sector organizations and for those in national bodies whose objective is to strengthen the relationship between sustainable development and the reputation of organizations (i.e., reputation as a result of promoting and putting into practice the values of the ethical standards of the organization, which is reflected in the ethical commitment of the employees). To improve their reputation and be perceived by the community as oriented towards sustainable development, public and private companies must first strengthen their ethical and sustainable culture through concrete actions based on codes of ethics and integrity unanimously accepted and understood by their employees.

Organizations which through their field of activity represent a potentially high risk of environmental pollution must use codes of ethics and integrity as tools to prepare their employees to face, successfully, any internal and external challenges that could harm both the organization and the environment. Therefore, it is imperative for the managers involved in energy, oil, and gas production and supply to not only understand the significance of promoting ethical and sustainable principles but also to actively encourage, through their own personal conduct, the demonstration of green ethical behavior by their employees. This ethical and green leadership can serve as a powerful catalyst in fostering a culture of sustainability within oil and gas sector organizations.

### 6.3. Limitations and Future Research

Our study is subject to a series of limitations. One of its limitations is the sample of the respondents from different branches of companies in the oil and gas sector, which represents a limitation of the degree of generalization of the research results. The sample’s structure is dominated by men and people with more than 15 years of experience in their organization. If concerning the preponderance of men, this limitation is justified by the specific nature of the field of activity, the age of the respondents, which is correlated with the length of time they have spent in the organization, determines us to continue our research and analyze the relationship between age, experience in the industry, and the predisposition of employees to manifest green ethical behavior.

Another limitation is the use of a quantitative method. However, in the future, it would be helpful to combine quantitative methods with qualitative ones based on interviews and even longitudinal studies to put employees in the position to make decisions that combine their green, ethical behavior with their values.

Future research will analyze the relationship between oil and gas sector organizations’ reputation, their culture, and the factors that motivate employees to demonstrate green, ethical behavior and continuously improve their performance. Until now, this relationship has been approached from a leadership perspective (Robertson & Barling, 2013; Solinger et al., 2020), but we will approach it from the perspective of the employees, especially employees who do not feel emotionally committed to the organization.

Finally, we emphasize the need for the concerted effort of all stakeholders to reduce the negative impact that pollution, which is the result of organizations’ activities, has on the environment. In this context, it is relevant for studies of green behavior in employees are carried out by analyzing the factors that directly impact their affective commitment and green behavior.

## Figures and Tables

**Table 1:** Descriptive profile of the sample.

Variable	N	%	Mean	Standard Deviation
Gender	319	100	1.24	0.425
Male	244	76.5		
Female	75	23.5		
Age	319	100	3.20	0.864
20–30 years	20	6.3		
31–40 years	33	10.3		
41–50 years	129	40.4		
51–60 years	137	42.9		
Category	319	100	1.71	0.454
Managing position	92	28.8		
Execution function	227	71.2		
Seniority in the organization	319	100	3.58	1.473
0 < 1 year	37	11.6		
1–5 years	59	18.5		
5–10 years	42	13.2		
10–15 years	45	14.1		
>15 years	136	42.6		

**Table 2:** An assessment of the composite reliability, convergent validity, and discriminant validity.

Variable	Codification of the Items	Factor Loading	Cronbach’s α	CR	AVE
Employees’ green ethical behavior			0.944	0.944	0.856
	EEGB1	0.927			
	EEGB2	0.940			
	EEGB3	0.928			
	EEGB4	0.905			
Evaluation of the degree of knowledge by employees of the particularities of the ethical values manifested in the organization			0.941	0.941	0.894
	EKE1	0.941			
	EKE2	0.951			
	EKE3	0.945			
The importance of the ethical values assumed by the organization through the code of ethics and integrity of employees			0.897	0.910	0.663
	IEV1	0.883			
	IEV2	0.734			
	IEV3	0.901			
	IEV4	0.723			
	IEV5	0.766			
	IEV6	0.861			
Evaluation of the degree of compliance with the rules of conduct within the organization			0.941	0.945	0.709
	RCC1	0.813			
	RCC2	0.783			
	RCC3	0.882			
	RCC4	0.857			
	RCC5	0.762			
	RCC6	0.898			
	RCC7	0.887			
	RCC8	0.843			
Assessing how compliance with the rules of conduct is reflected in sustainable development			0.881	0.884	0.808
	RCSD1	0.909			
	RCSD2	0.925			
	RCSD3	0.861			

Source: Smart PLS software: SmartPLS.

**Table 3:** Hypothesis testing.

Hypotheses	Original Sample (O)	Sample Mean (M)	Standard Deviation (STDEV)	T Statistics (|O/STDEV|)	*p* Values	STATUS
H1: EEGB -> RCSD	−0.367	−0.375	0.173	2.122	0.034	Approved
H2: EKE -> EEGB	0.733	0.731	0.037	19.835	0.000	Approved
H3: EKE -> IEV	0.878	0.879	0.015	57.285	0.000	Approved
H4: EKE -> RCC	−0.340	−0.346	0.115	2.945	0.003	Approved
H5: IEV -> EEGB	0.282	0.285	0.041	6.874	0.000	Approved
H6: IEV -> RCC	0.922	0.927	0.105	8.743	0.000	Approved
H7: IEV -> RCSD	0.936	0.943	0.162	5.778	0.000	Approved
H8: RCC -> EEGB	−0.047	−0.047	0.021	2.246	0.025	Approved

Source: Smart PLS software: SmartPLS.

**Table 4:** The mediation between variables in the model.

Mediation	Original Sample (O)	Sample Mean (M)	Standard Deviation (STDEV)	T Statistics (|O/STDEV|)	*p* Values
EKE -> IEV -> RCC	0.810	0.815	0.096	8.419	0.000
EKE -> IEV -> RCSD	0.822	0.830	0.148	5.545	0.000
EKE -> IEV -> EEGB	0.248	0.250	0.037	6.726	0.000
EKE -> EEGB -> RCSD	−0.269	−0.273	0.125	2.146	0.032
IEV -> EEGB -> RCSD	−0.104	−0.108	0.055	1.880	0.060
RCC -> EEGB -> RCSD	0.017	0.019	0.014	1.251	0.211
EKE -> RCC -> EEGB	0.016	0.017	0.010	1.623	0.105
IEV -> RCC -> EEGB	−0.043	−0.044	0.021	2.063	0.039

Source: Smart PLS software: SmartPLS.

## Data Availability

The data that support the findings of this study are available from the corresponding author upon reasonable request.
